# Post-renal Transplant Pseudoaneurysm With Renal Artery Stenosis: A Rare and Life-Threatening Complication

**DOI:** 10.7759/cureus.87117

**Published:** 2025-07-01

**Authors:** Bunnarin Theng, Kanishka V Chelikani, Aparna Medarametla, Laura Jacobson

**Affiliations:** 1 Department of Radiology, University of Texas Medical Branch at Galveston, Galveston, USA

**Keywords:** ct scan, pseudoaneurysm, renal artery stenosis, renal transplantation, ultrasound

## Abstract

This case report follows a 64-year-old male patient who underwent deceased-donor renal transplantation for end-stage renal disease secondary to chronic hypertension and type 2 diabetes mellitus. On presentation, he displayed signs of volume overload, significant weight and blood pressure increases, and minimal urine output. Initial ultrasound revealed elevated peak systolic velocity at the transplant kidney anastomotic site, suggesting post-transplant renal artery stenosis along with a larger-than-expected iliac artery concerning for pseudoaneurysm, later confirmed with subsequent CT scan findings. This case outlines the rare nature of pseudoaneurysm post-renal transplant and emphasizes the efficacy of CT as a tool for diagnosis when ultrasound findings are equivocal.

## Introduction

In a typical kidney transplant, the donor kidney is placed in the iliac fossa outside the peritoneal cavity, with its blood vessels connected to the recipient’s external iliac vessels and the ureter attached to the bladder to restore urinary function [1]. However, renal transplantation can lead to a variety of postoperative vascular complications, which may include arteriovenous fistula, pseudoaneurysm, graft thrombosis, transplant renal artery stenosis, or stenosis of the conduit iliac artery [2]. Pseudoaneurysms in kidney transplants are rare, occurring in less than 1% of cases [3]. However, pseudoaneurysms pose significant risks, as they can suddenly rupture and result in life-threatening hemorrhage, functional impairment, transplant loss, or even death if not identified and treated promptly [3]. This case report highlights a rare occurrence of pseudoaneurysm with concurrent renal artery stenosis following kidney transplantation.

## Case presentation

This is the case of a 64-year-old male with end-stage renal disease secondary to chronic hypertension and type 2 diabetes mellitus. He had been on hemodialysis for six years before receiving a deceased donor kidney transplant. Postoperatively, he developed delayed graft function, evidenced by persistent oliguria despite diuretics therapy, elevated blood urea nitrogen and serum creatinine (above 12 mg/dL), hyperkalemia resistant to medical management, and anemia requiring multiple blood transfusions. Ultrasound showed no hydronephrosis, perinephric fluid collection, obstructive or vascular abnormalities, supporting intrinsic graft dysfunction as the likely cause. He required three hemodialysis sessions in the hospital and by discharge, he had stable urine output and downtrending creatinine to 10.52 mg/dL. He remained hemodynamically stable, tolerated oral intake, and was discharged on prednisone, tacrolimus, and mycophenolate with close outpatient follow-up.

Two months later, he showed signs of volume overload, including a 12-pound weight gain, elevated creatinine and blood pressure to 197/91, and multiple days of anuria. Physical exam showed 3+ pitting edema and fine tremors but no abdominal tenderness, altered mental status, or signs of infection. He was readmitted for evaluation due to concerns for allograft dysfunction and was started on high-dose IV diuretics with minimal response. He was transferred to the ICU and underwent urgent dialysis with ultrafiltration. Empiric antibiotics were initiated with ertapenem 500 mg IV once daily and amoxicillin 500 mg orally twice daily for targeted coverage of *Enterococcus faecalis* identified on urine culture. He was also started on pulse-dose steroids 500 mg for three days for suspected rejection.

**Figure 1:**
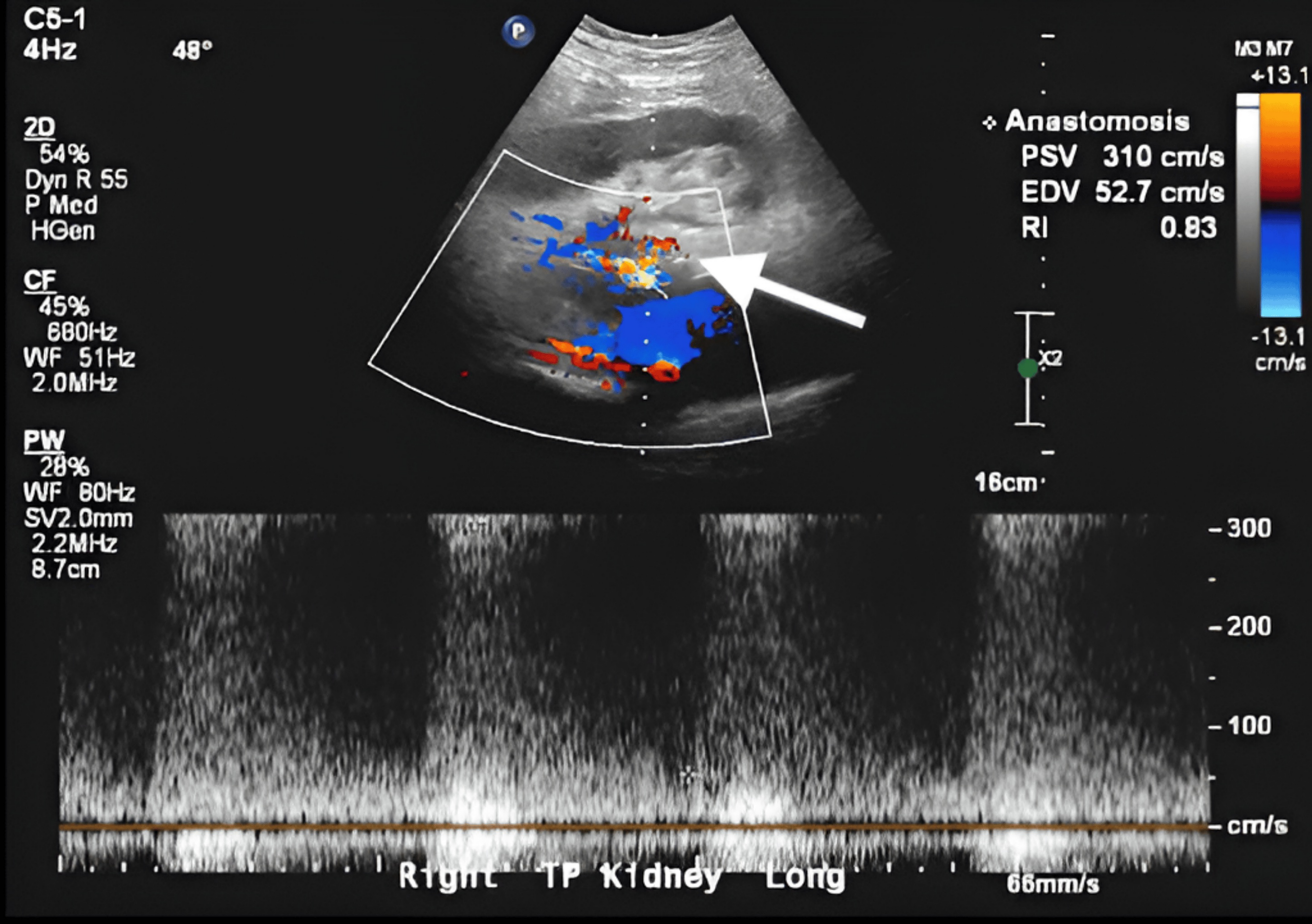
Elevated PSV at anastomosis (310 cm/s), suggestive of transplant renal artery stenosis (white arrow) PSV: peak systolic velocity; EDV: end diastolic velocity; RI: resistive index; CF: color flow; WF: waveform; PW: pulsed wave; TP: tranverse plane

A Doppler ultrasound revealed a transplanted kidney measuring 10 x 4.4 x 7.7 cm compared to 14 x 6.2 x 6.6 cm from a previous study two months ago with interval development of mild to moderate hydronephrosis. There was evidence of elevated peak systolic velocity at the anastomotic site at 310 cm/s compared to 155 cm/s previously, concerning for possible stenosis (Figure 1).

**Figure 2:**
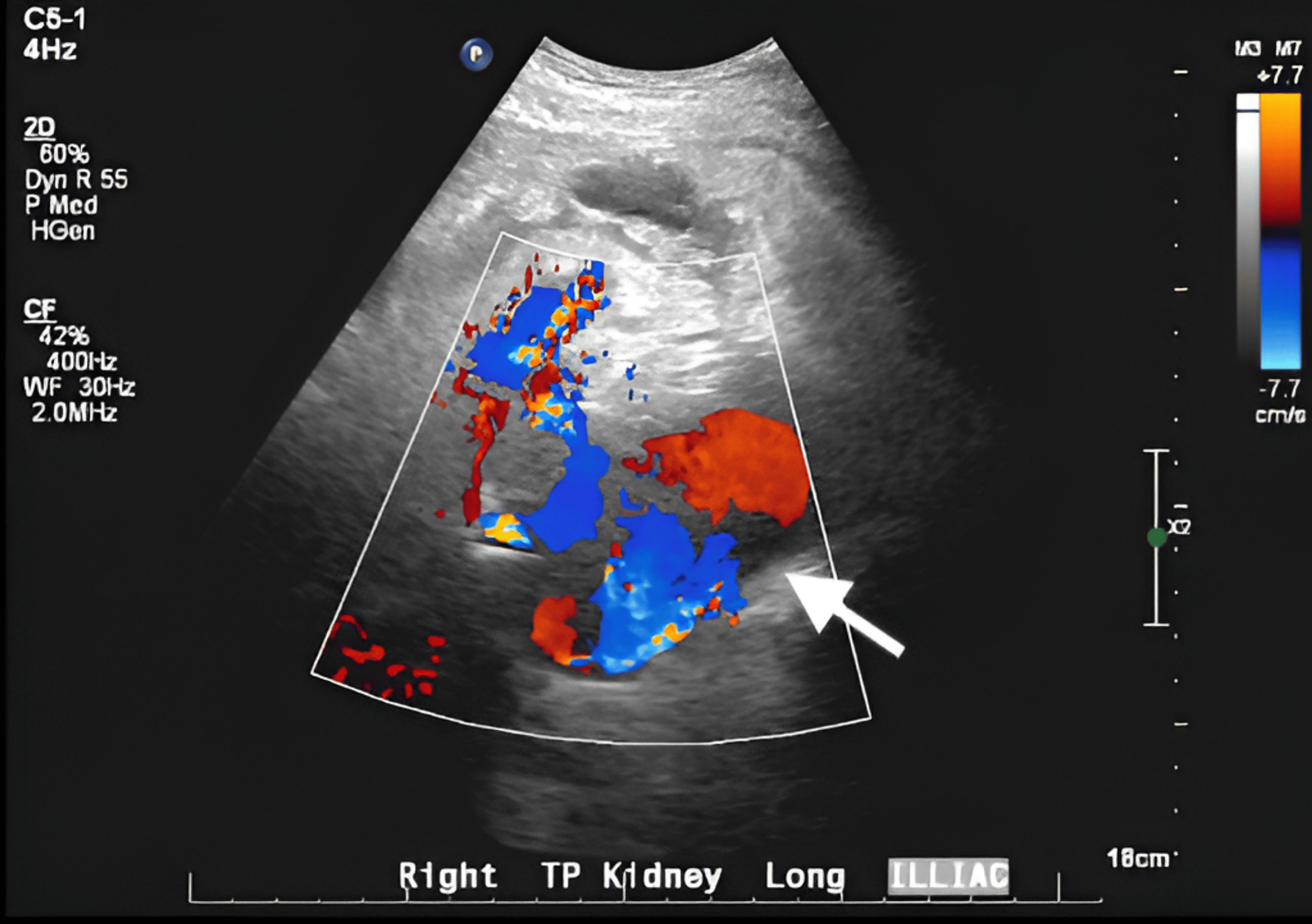
A pseudoaneurysm sac (white arrow) medial to the renal hilus near the region of the iliac artery CF: color flow; WF: waveform; TP: transverse plane

A 3 cm hypoechoic structure lateral to the transplanted kidney was also seen, which was concerning for perinephric fluid collection or a hematoma. An additional hypoechoic structure was seen medial to the renal hilus near the region of the iliac artery which demonstrated internal color flow and Doppler waveform (Figure 2). It was identified as the iliac artery but is larger than expected for a normal iliac artery and appears larger compared to the previous scan. This was concerning for possible aneurysm or pseudoaneurysm, and a CT scan was recommended for further evaluation. 

**Figure 3:**
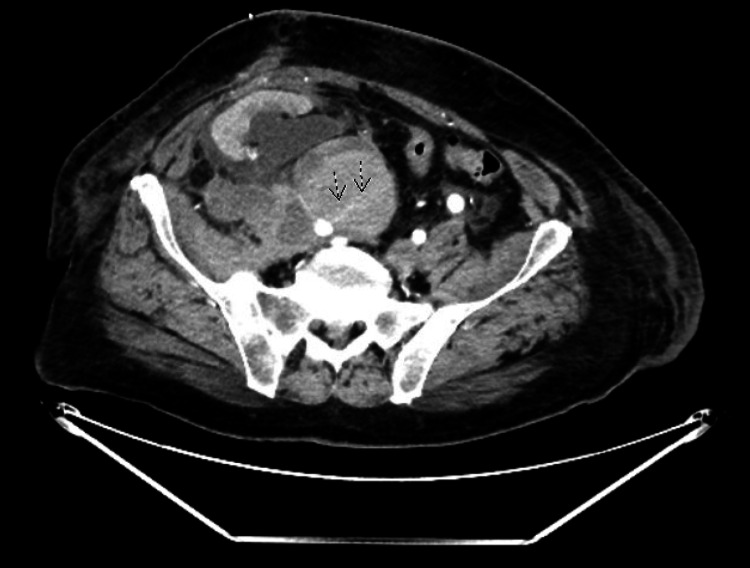
Axial view of pseudoaneurysm compressing on transplanted renal artery (striped arrows)

**Figure 4:**
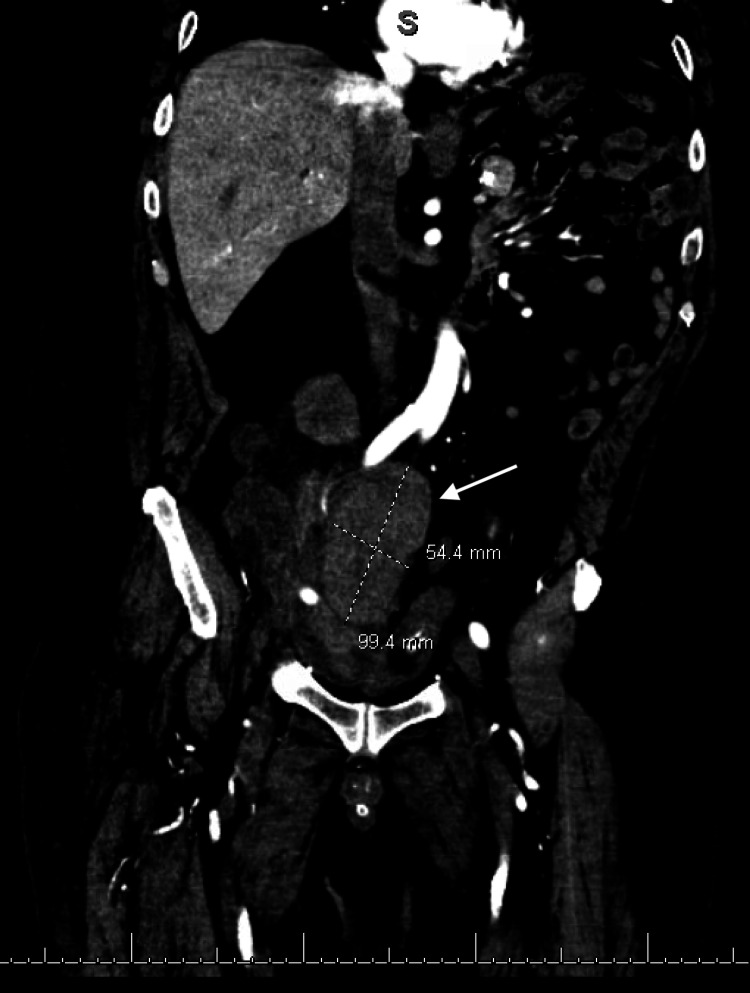
Coronal view of pseudoaneurysm, measuring 10 x 5.4 cm (white arrow)

A follow-up CT scan of the abdomen and pelvis with and without contrast showed hyperdensity anteromedial to the right external iliac artery measuring 10 x 5.4 cm with attenuation following the blood pool, most consistent with a pseudoaneurysm at the level of the transplant renal artery, resulting in a significant mass effect on the transplant renal artery (Figures 3, 4).

**Figure 5:**
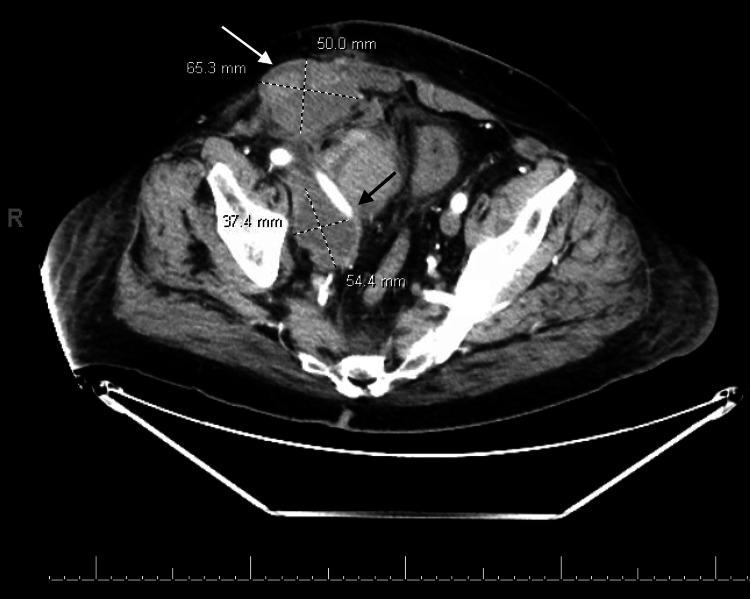
A hematoma involving the right pelvic wall measuring 5.5 x 3.7 cm (black arrow) and a hematoma involving the right rectus measuring 6.5 x 5 cm (white arrow)

There was also evidence of moderate to severe hydronephrosis of the transplanted kidney and a high-density lobulated structure noted in the right pelvic wall measuring 5.5 x 3.7 cm (Figure 5), likely representing a hematoma. Similarly, an additional lobulated high-density lesion is noted involving the right rectus measuring 6.5 x 5 cm, likely representing another hematoma (Figure 5). 

He then underwent an exploratory laparotomy with evacuation of retroperitoneal hematoma, ligation of the right external iliac artery pseudoaneurysm, and placement of an aortic bypass graft using a 10 mm Dacron conduit. Postoperatively, his urine output improved significantly and dialysis was no longer required. He was discharged with a prolonged course of IV antibiotics via a peripherally inserted central catheter (PICC) line, including ceftriaxone, metronidazole, and fluconazole, per infectious disease guidance for presumed mycotic pseudoaneurysm and a close outpatient follow-up. He remained clinically stable following discharge with improving metabolic acidosis and stable hemoglobin with improved graft function.

## Discussion

A pseudoaneurysm occurs when an arterial wall is punctured, leading to the pooling of blood in an outpouching bound by a barrier made of coagulation proteins [4]. Also known as a false aneurysm, this pathology differs from a true aneurysm in that it does not involve ballooning of all three layers of the vessel wall. The etiology of pseudoaneurysms may be due to iatrogenic causes such as complications of arterial catheterizations, percutaneous hemodialysis, loss of integrity of surgical arterial anastomoses and central line placements, or due to non-iatrogenic causes such as infection, trauma, malignancy, atherosclerosis, or vasculitis [5,6]. Risk factors for developing an iatrogenic pseudoaneurysm include hypertension, atherosclerosis, use of anticoagulant medications, female sex, and systemic disease processes that delay the healing process, such as diabetes [4]. Pseudoaneurysms of the iliac artery are extremely rare and generally asymptomatic, though the risk for rupture and subsequent hypovolemic shock calls for timely identification and repair [6]. Renal transplant procedures most commonly place the transplanted kidney in the recipient’s pelvis, with renal vein and artery anastomoses to the external iliac vessels, making this region especially susceptible to vascular complications [1]. Although rare, most transplant-related pseudoaneurysms occur within the first three months following surgery, particularly those of infectious origin, although it can take months up to years later in idiopathic or slowly evolving cases [3].

Vascular complications post-kidney transplant have an incidence rate of 6-30% and include renal artery stenosis, arterial/venous thrombosis, arteriovenous fistula, and renal artery pseudoaneurysm [7]. Pseudoaneurysms post-renal transplant are quite rare, with an estimated incidence of less than 0.5% and they may involve the transplant renal artery, the external iliac artery, or the anastomotic site between the two vessels, as in our case [3].

Various etiologies exist for pseudoaneurysm formation post-renal transplant and can be grouped into infectious causes, such as mycotic and bacterial infection, and noninfectious etiologies, including iatrogenic vessel wall injury, defective vascular reconstruction techniques, and chronic rejection [3,7]. Infective pseudoaneurysms secondary to mycotic infections are more frequent and may present at multiple sites along the vascular reconstruction. Pseudoaneurysms related to defective techniques are typically present at the site of anastomosis [8].

Presentation of post-renal transplant pseudoaneurysm varies with the size of the pseudoaneurysm, with smaller aneurysms being asymptomatic and diagnosed incidentally [9]. Larger pseudoaneurysms may present with kidney dysfunction, such as in the case we present, where mass effect from the pseudoaneurysm resulted in transplant renal artery stenosis, leading to a presentation consistent with kidney injury. Other presentations include pain at the transplant site that may be associated with a pulsatile mass, malignant hypertension, and lumbar plexopathy due to compression by the pseudoaneurysm [3,8].

These presentations raise concern for a post-renal transplant pseudoaneurysm, which typically leads to an ultrasound. Generally, the main imaging modality used to diagnose a suspected pseudoaneurysm is a real-time duplex ultrasound (US), sometimes followed by a computed tomography angiogram (CTA) or magnetic resonance angiogram (MRA) to better evaluate the surrounding anatomy [4,5]. Duplex US has a sensitivity of 100% and is useful in determining the size, origin, and velocity of flow in the pseudoaneurysm, which generally appear as anechoic structures in B-mode. A characteristic “yin-yang” sign depicting bidirectional flow can be identified on the color Doppler US, which represents the pulsatile flow of arterial blood into and out of the pseudoaneurysm sac with systole and diastole, respectively. Ultrasound is also used to evaluate for the presence of thrombosis, which can present with varying degrees of echogenicity depending on when the thrombus initially formed. In addition to imaging the sac, the neck of the pseudoaneurysm is also examined, and the surrounding structures are surveyed for extravasation of arterial blood and potential hematoma formation. Digital subtraction angiography (DSA) is essential not only for accurately diagnosing renal pseudoaneurysms by providing detailed vascular imaging but also for enabling minimally invasive treatments like coil embolization or covered stent placement, which have greatly lowered the risks associated with surgery [10]. In our case, the initial ultrasound depicted elevated peak systolic velocity at the anastomotic site concerning renal artery stenosis. The precise structure causing the compression could not be fully characterized; however, a CT scan with and without contrast confirmed an external iliac artery pseudoaneurysm.

Our clinical scenario is similar to a case series from Fananapazir et al. in which ultrasound accurately depicted transplant renal artery stenosis, but performed poorly when detecting the pseudoaneurysm itself [11]. Ultrasound is operator-dependent, has difficulty depicting the anastomotic region, and bowel gas can obscure the area of interest. Therefore, CTA or MRA plays an important role in subsequent imaging when a pseudoaneurysm is suspected and can aid in pre-procedural planning [11]. On CTA, pseudoaneurysms present as hyperattenuating masses adjacent to the source artery [12]. Non-contrast MRA is done instead when there are contraindications to using iodinated contrast agents to visualize the pseudoaneurysm and blood flow within and around the sac. On non-contrast MRA, pseudoaneurysms present as hypointense moieties with subtle delineation from the surrounding vascular structures.

Management of post-renal transplant pseudoaneurysms is complex and requires a multidisciplinary approach, taking into account the patient’s needs. It is generally accepted that pseudoaneurysms greater than 2.5 cm are at high risk of rupture and must be repaired, while smaller aneurysms less than 2.0 cm can be surveilled with serial imaging to monitor for spontaneous resolution [7]. Interventions include minimally invasive procedures such as percutaneous thrombin injection under ultrasound guidance or an endovascular approach with embolization or stent-grafting [3]. Open surgery is considered the best option in cases where hemodynamic instability is present and emergent surgical exploration is necessary [11]. This method requires vascular reconstruction, however, which may cause renal ischemia and loss of the graft, and may require the need of repeat surgery [8]. Thus, for most cases, minimally invasive endovascular repair of the pseudoaneurysm is preferred, although it may be technically challenging due to angulation of the anastomosis [3].

## Conclusions

Pseudoaneurysm formation at the transplant renal artery anastomosis, particularly when accompanied by renal artery stenosis, presents a rare but serious threat to graft function and patient stability, which should be considered in transplant recipients with unexplained volume overload and declining urine output. Cross-sectional imaging, particularly CT or CTA, is critical for diagnosis when ultrasound findings are limited, and timely endovascular management may prevent irreversible graft damage or the need for nephrectomy.
